# The diel activity pattern of mountain hare (*Lepus timidus*) on managed heather moorland in Scotland

**DOI:** 10.1002/ece3.7512

**Published:** 2021-05-01

**Authors:** Graham W. Pettigrew, Valentina Di Vita, Maxine Pettigrew, Jason S. Gilchrist

**Affiliations:** ^1^ School of Applied Sciences Edinburgh Napier University Edinburgh UK

**Keywords:** activity, camera trap, crepuscular, diel, *Lepus timidus*, mountain hare

## Abstract

The research presented in this paper provides an insight into the behavioral ecology of mountain hares on heather moorland in the Lammermuir Hills of southeast Scotland. We examine the seasonal and diel activity patterns using camera traps over a period of 12 months. The rate of camera detections was calculated for the different divisions of the 24‐hr cycle (daylight, dusk, night, and dawn). During autumn and winter (October–February), the activity pattern was crepuscular with greater activity at dusk than at dawn. Daylight activity was relatively low, and there was a regular pattern of small peaks of activity during the night. In spring and summer (March–September), peaks of crepuscular activity remained evident but daylight activity was much more prevalent than during autumn and winter, and night activity was lower. We discuss the problematic definition of twilight and present an explanation for seasonal changes in the pattern of diel activity that is linked to the reproductive cycle of the mountain hare.

## Graphical abstract

Mountain hare activity in the south of Scotland during autumn and winter was crepuscular with greater activity at dusk than at dawn. Daylight activity was relatively low and night activity showed an episodic pattern. In spring and summer, peaks of crepuscular activity remained evident but daylight activity was much more prevalent, and night activity was lower.

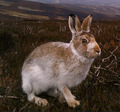

## 1 INTRODUCTION

The mountain hare (*Lepus timidus*) is a native species of the high alpine regions of the Cairngorm Mountains of Scotland (Watson, 2013). During the 19th century, animals were translocated from there onto heather moorland that was managed for game shooting (Flux, 1970). These open alpine and heather moorland habitats contrast with a preference for boreal habitat across much of the extensive range of the species in northern Europe and Asia (Flux, 1970) although mountain hares are also found on tundra and agricultural land (Angerbjörn & Flux, 1995). Mountain hares breed between February and September (Angerbjörn & Flux, 1995). They can be present at densities of up to 80 km^−2^ in their native alpine habitat in Scotland (Watson, 2013) and even higher densities of up to 245 km^−2^ on heather moorland (Watson & Hewson, 1973) where the encouragement of new heather growth and the control of predators are part of the management regime (Hesford et al., 2019). In recent years however, extensive culling of mountain hares on shooting estates has been associated with some local declines (Watson & Wilson, 2018).

The hill habitats in Scotland can be unforgiving in winter with high winds, low temperatures, and lack of shelter. Mountain hares do not hibernate, nor burrow, and they spend their resting time in superficial depressions called forms usually in tall heather. These locations may afford some shelter during adverse weather conditions (Flux, 1970; Thirgood & Hewson, 1987). They have a thick white winter pelt that serves the dual purpose of insulation and camouflage and is molted in spring (Flux, 2009; Hewson, 2009). In winter, the hare adapts its diet toward vegetation that may still be accessible above snow cover or can be reached by digging (Hewson, 1962; Hulbert et al., 2001).

Diel (24‐hr cycle) activity studies are of importance in understanding the rhythms of mammalian life and the factors that influence and control these rhythms. Many studies have shown that mountain hares are predominantly crepuscular/nocturnal animals (reviewed in Angerbjörn & Flux, 1995) but the details of their circadian rhythms and seasonal variations are not well defined. Camera traps are a useful tool in recording profiles of animal activity (reviewed in Rowcliffe et al., 2014), particularly for animals such as mountain hares that are predominantly nocturnal and that live in remote habitats. Ikeda et al. (2016) and Ogurtsov et al.  (2018) used camera trapping to study diel activity and seasonal changes in their studies of mountain hares in the boreal forests of Japan and Russia, respectively.

Counting mountain hare is much easier when they are active. Using part of the same dataset presented here, one of us has explored how activity studies can inform the best time of year and the best time in the 24‐hr cycle to count (Pettigrew, 2020): this at a time when there is controversy surrounding uncontrolled culling (Brooker et al., 2018).

This study examined the activity of mountain hares in the Lammermuir Hills of southeast Scotland. We used camera trapping to explore in detail the patterns of circadian rhythm and its seasonal variations. The data presented inform our knowledge of the natural history of this native mammal species.

## 2 MATERIALS AND METHODS

### 2.1 Study area

**FIGURE 1:**
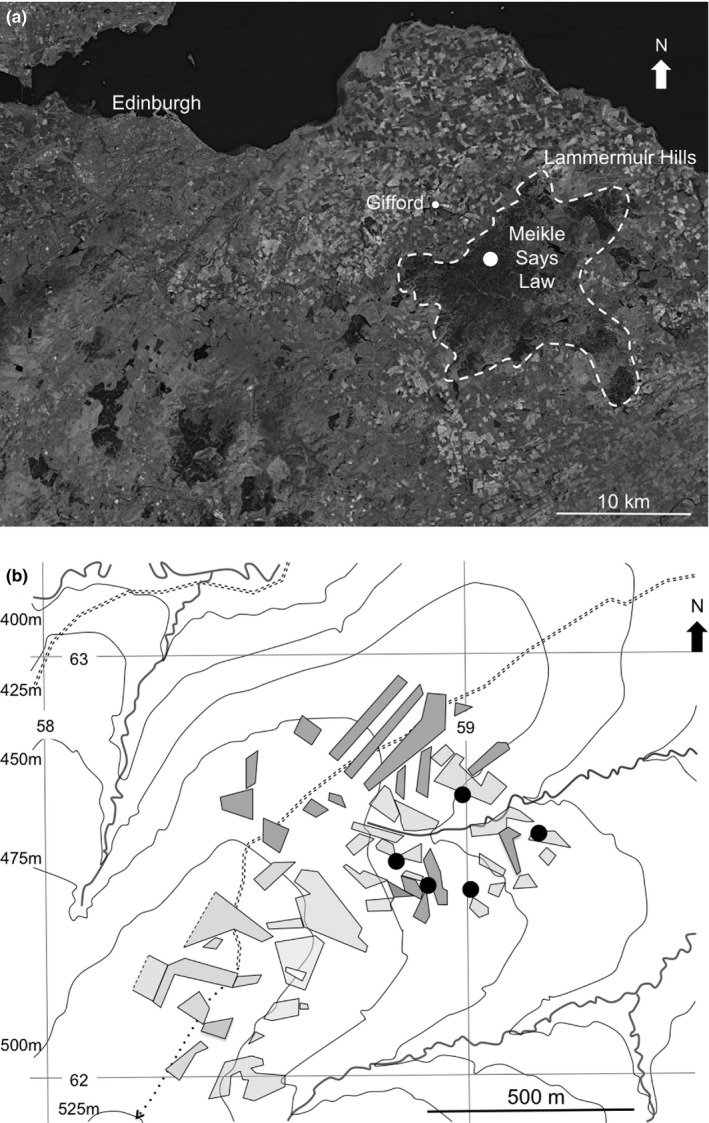
The mountain hare study area. (a) Satellite view of the general area of the Lammermuir Hills in south‐east Scotland. These hills are indicated by the broken line, and the study area is shown as a white circle. (Microsoft product screen shot reprinted with permission from Microsoft Corporation). (b) The study area is amplified to show the five camera positions (black circles). The new muirburn of 2019 is shown in dark gray, and older muirburn is shown in light gray

The study was undertaken within a 1 km square area of heather moorland managed for driven grouse shooting on the eastern slopes of Meikle Says Law, Lammermuir Hills, Scotland (SW corner of 1 km square study area: Latitude 55.813758, Longitude −2.607933). The general topography of the study area, the pattern of muirburn (Yallop et al., 2006), and the location of camera traps are shown in Figure 1b. During mountain hare surveys (Pettigrew, 2020), up to 20 individual hares have been routinely counted by the Lammermuir Hare Group in the camera study area.

### 2.2 Camera traps

Five camera traps (AUCEE 12 MP H9 Hunting Cameras) were set at 0.7 m above ground level on posts with a 5–10 degree downward tilt at the locations shown in Figure 1b between October 2018 and September 2019 (8,760h). We selected nonrandom locations to maximize total detection rates, by avoiding placing cameras in deep heather where they would be unlikely to record hare movement. Three of the locations were regenerating heather areas with associated hare runs and two were dry gullies with a mixture of heather, grasses, and rushes. A pilot study found 184 hare detections for 5 nonrandom cameras in a single month but only 32 hare detections for 5 random cameras (Pettigrew & Di Vita, unpublished data). Maximizing detections contributes to the smoothness of activity profiles and the confidence that can be placed in their key features (Lashley et al., 2018). The cameras were separated by at least 50 m (exceeding the detection limit of the infrared cameras (Brown & Gehrt, 2009) and they were orientated pointing away from nearest neighbors in order to reduce the likelihood of double counting.

The cameras were set on Greenwich Mean Time throughout the year and took 3 consecutive still images after a motion trigger, followed by a delay of 10 s. Images were 8 MP. Infrared detection distance was set to ‘far’, and motion sensitivity was set to ‘medium’. Camera trap images were downloaded every 2 weeks and camera times were reset if they had incurred time errors (which were less than 6 min). For each image that contained a mountain hare, we evaluated whether or not it represented an independent event. It is common for camera trap studies to apply an arbitrary threshold duration between photos to assign independence of events. Our general rule was that camera triggers separated by less than 2 min were not counted as independent events and those separated by more than 2 min were counted as independent events. However, we have three exceptions to the general rule. Multiple hares in one image and two visually distinctive hares in different images with triggers within 2 min are counted as independent events. Triggers caused by the same hare (based on visual appearance and positional information) which extend beyond 2 min are rejected.

In our dataset of 2,054 events, there are 192 instances of multiple hares within one image; there are 49 instances of two different hares appearing in different images within 2 min; and there are 45 instances of the same hare triggering the camera at intervals of >2 min. The image data and applications of exceptions to the 2‐min general rule are available in the supporting files S1a and S1b, respectively, linked to from the Data Accessibility Statement. [Correction added on 24 May 2021 after first publication: the location of the links to the supporting files has been clarified in the preceding sentence.]

For analysis of the independent events (subsequently called ‘detections’), we define a 2‐hr postsunset (dusk) period and a 2‐hr presunrise period (dawn). We defined daylight as between sunrise and sunset, and night as between the end of the dusk period and the beginning of the dawn period. The times of sunset and sunrise and the hours of daylight and night were calculated for each day using the data tables at https://www.thetimeandplace.info/uk/ for the nearby village of Gifford (Figure 1a). The time stamps for detections were tabulated in Excel, categorized as morning (before 12:00) and afternoon (12:00 and after) and then re‐calculated as decimal time relative to either sunset (afternoon group) or sunrise (morning group). Vazquez et al. (2019) found that this ‘average anchoring method’ was the best means for reliable extraction of crepuscular activity at higher latitudes.

The detections from the five cameras for each day of a month were assigned to the divisions: daylight, dusk, night, or dawn. Daily detection rates for these divisions were calculated and then summed for the month. The monthly detection rates for each of the divisions of the day were expressed as a percentage to allow for the variations in the numbers of detections for the different months. An example of the calculation is available in supporting file S2, which is accessible via the link in the Data Availability Statement. [Correction added on 24 May 2021 after first publication: the location of the link to the supporting file has been clarified in the preceding sentence.]

In order to provide a rational basis for grouping the months of the year, we plotted an activity index for the daylight and night periods akin to that of Manly et al. (2002). For this specific analysis (in contrast to the definitions above), night is defined as the period between sunset and sunrise (i.e., it includes both the dusk and the dawn period). This is done to simplify the graph and focus on the activity changes in the daylight period.

Activity index=Proportion of detections in the daylight or night period/Proportion of time in the daylight or night period

On the basis of this analysis (see Results, Figure 3), we grouped the months into the autumn–winter period of October to February and the spring–summer period of March to September and we analyzed the detection patterns in blocks of 0.4 hr, a choice of time subdivision based on the relative smoothness of the profiles and its divisibility into the 2 hr dusk and dawn periods (see Results, Figure 4).

We tested for significant differences in hare detection rate using an ANOVA with monthly detection rate as the response variable and the divisions of the 24‐hr cycle (daylight, dusk, night, dawn) as the predictor variable. Separate ANOVA was applied to the October to February and March to September data. The analysis was conducted with the statistical software R (R Development Core Team, 2019). The distribution of residuals satisfied the Levene test for homogeneity of variance and the Shapiro test for normality. Pairwise comparison of divisions of the 24‐hr cycle was done by the Tukey *post hoc* test. A further ANOVA was applied to compare the divisions of the 24‐hr cycle for all 12 months of the calendar year.

## 3 RESULTS

**FIGURE 2:**
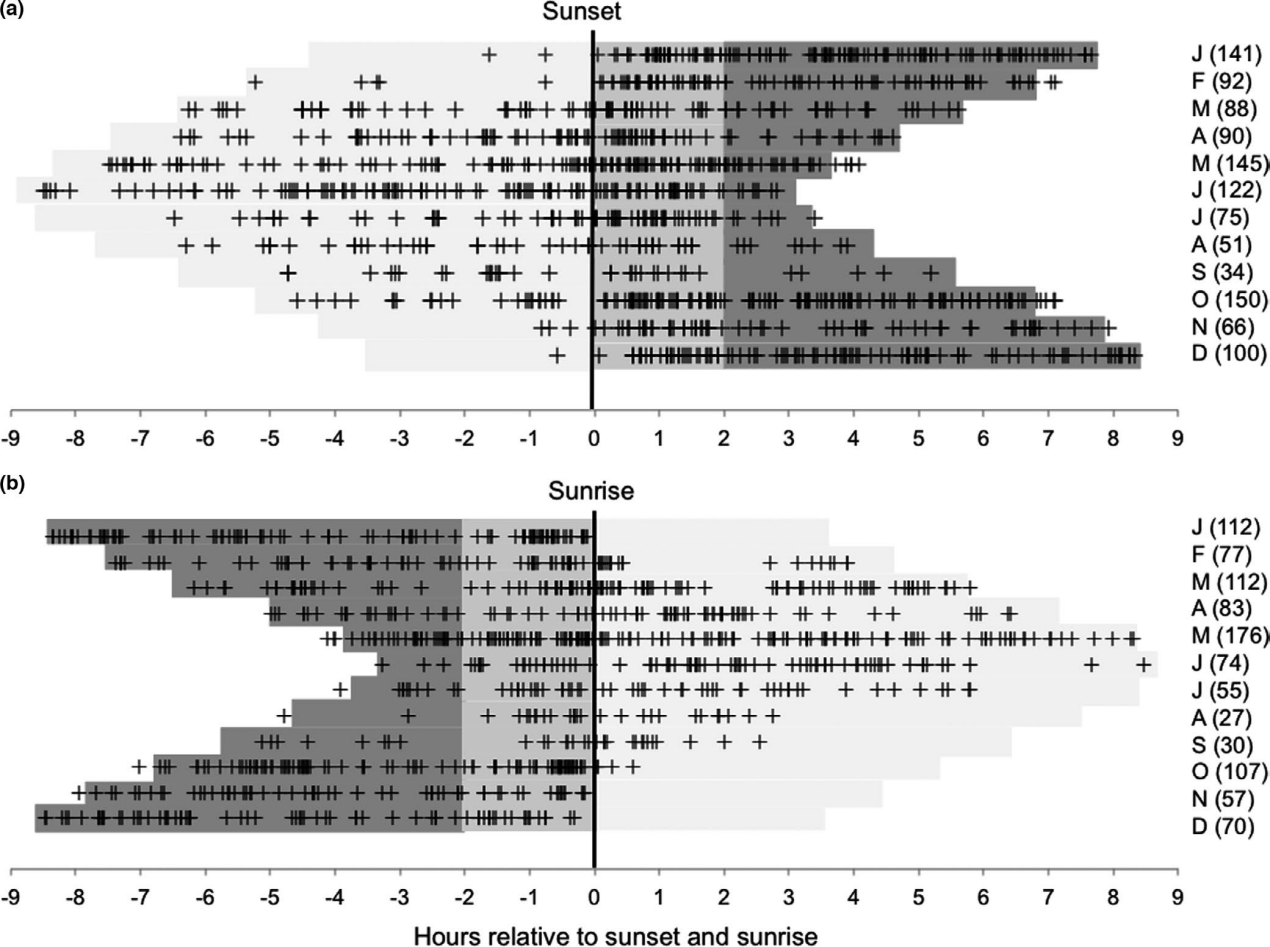
The diel patterns of mountain hare activity over 12 months. (a) Detections relative to the time of sunset (black vertical line) in decimal hours on the horizontal axis. (b) Detections relative to the time of sunrise (black vertical line) in decimal hours on the horizontal axis. In both (a) and (b), the 12 months are arranged from top to bottom from January to December with the number of detections shown. In both (a) and (b), light shading represents daylight, mid‐gray shading represents dawn or dusk, and dark gray shading represents night. In an individual month, the horizontal combination of gray shading represents the 12‐hr period between noon and midnight (a) or between midnight and noon (b). Some data points fall outside these bars because the bars are positioned at the average point for each month

**FIGURE 3:**
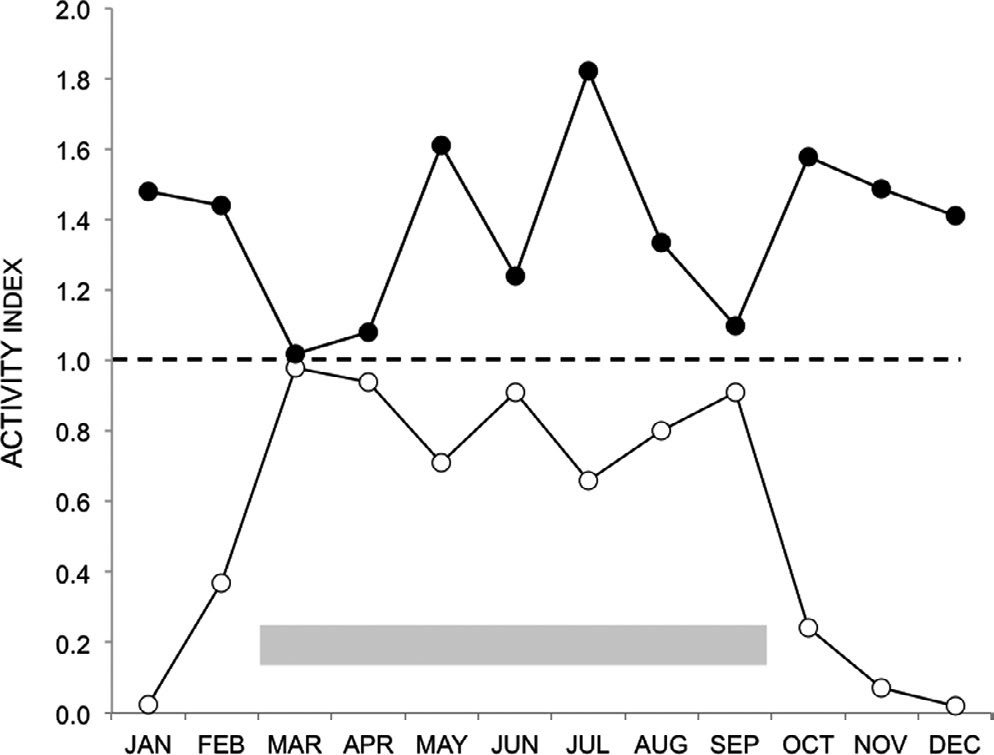
Levels of daylight and night activity of the mountain hare in the different months of the year. Activity index = Proportion of detections for the daylight or night periods (open circles and closed circles, respectively)/Proportion of time for the daylight or night periods. For this specific purpose, ‘night’ is defined as the period between sunset and sunrise (ie it includes both the dusk and the dawn period). The activity index was calculated for each month of the year. A value of one for that index (broken line) indicates that the proportion of detections and the proportion of time for that division of the 24‐hr cycle are equal. The months between March and September indicated by the gray‐shaded area are associated with values for the daylight activity index (open circles) that approach a value of 1. This is in contrast to the low values for the daylight activity index found for the months of October to February

**FIGURE 4:**
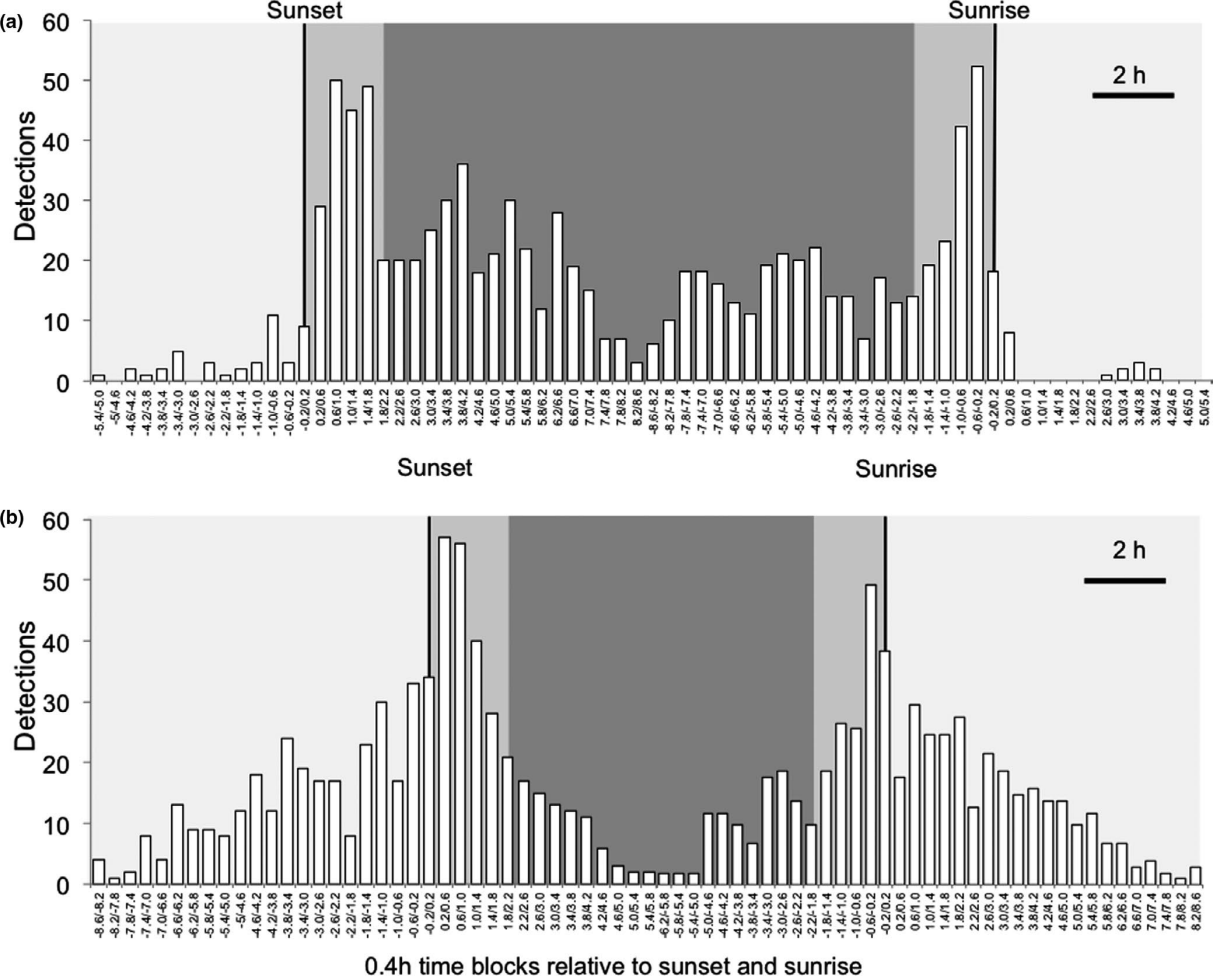
Mountain hare detections organized in 0.4 hr blocks over the 24‐hr cycle and grouped for autumn–winter (a) and spring–summer (b). (a) The autumn–winter period includes the months of October–February. The total number of detections in this period was 972. (b) The spring–summer period includes the months of March–September. The total number of detections in this period was 1,082. In both (a) and (b), light shading represents daylight, mid‐gray shading represents dawn or dusk, and dark gray shading represents night. The left vertical line represents sunset, and the right vertical line represents sunrise

**FIGURE 5:**
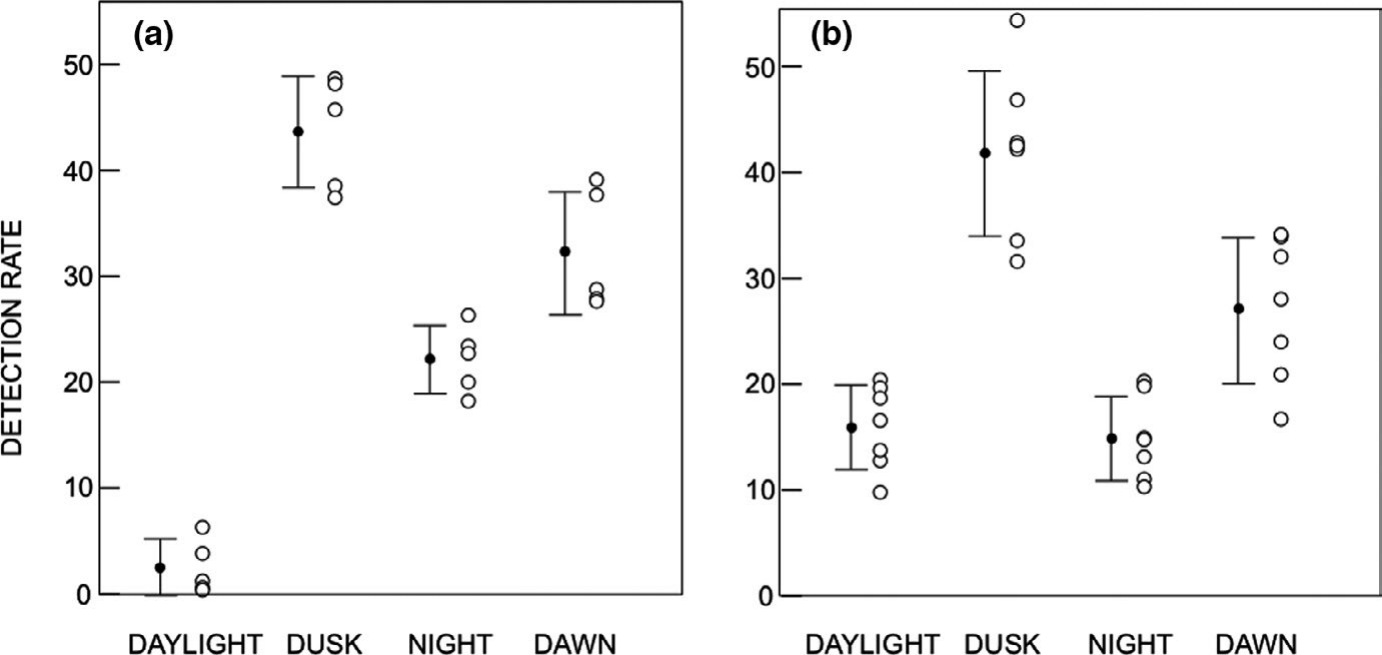
Mountain hare detection rates during daylight, dusk, night, and dawn over the 5 months of October–February (a) and the 7 months of March–September (b). The closed circles represent the means and the vertical bars the standard deviation

The analysis of the results from camera trapping follows a step‐by‐step sequence which corresponds to the sequence of Figures 2, 3, 4, 5. We present the raw data across the months of the year in Figure 2. We analyze the rise in daylight activity in spring and summer (Figure 3), and this allows us to group the data into two divisions of the year: March–September and October–February. We group the data into 0.4 hr blocks to show the diel activity patterns for the two divisions of the year (Figure 4). We show that these two divisions of the year have significantly different diel activity patterns (Figure 5).

Individual detections are shown in Figure 2 and summed for each month. During the spring and summer months of March–September, hares were consistently active in daylight (40%–65% of detections). In the autumn and winter months of October–February, there were much fewer daylight detections (1%–14%), and in the months November–January, there were no detections in the morning post‐sunrise period. Based on the activity index (Figure 3) and the pattern of Figure 2, we chose the divisions of the year that are used below with October and February having the appearance of transitional months.

During October–February (Figure 4a), there were sharp dawn and dusk activity peaks, very little daylight activity and activity during the night with small peaks separated by 1.5–2.5 hr. In March–September (Figure 4b), there were still peaks of activity during dawn and dusk but there was markedly greater activity during the day. This shift to daylight activity in spring–summer is not simply because of an increase in daylight hours. March and October have a similar partition of daylight and night but a very different pattern of daylight detections (Figures 2 and 3).

We found a significant difference in detection rate between the divisions of the 24‐hr cycle for October–February (*F*_3,16_ = 78.78, *p* < .001) and March–September (*F*_3,24_ = 32.08, *p* < .001). The Tukey post hoc test showed that, for October to February (Figure 5a), the mean detection rate was greatest for dusk, followed by dawn, night, and daylight with all differences significant (*p* < .01). For the March–September results (Figure 5b), the mean detection rate was highest for dusk which was significantly greater than that for dawn (*p* < .001). Dusk (*p* < .001) and dawn (*p* < .01) frequencies were significantly greater than those for night and daylight, but night and daylight frequencies showed no significant difference (*p* = .99).

In a separate ANOVA (*F*_7,40_ = 39.6, *p* < .001), the detection rate was also compared for the different divisions of the 24‐hr cycle between the two divisions of the year. Only the daylight division showed a significant difference (*p* = .002) between October–February and March–September.

On the basis of Figure 4 and the statistical analysis of Figure 5, we conclude that the activity that we see throughout the year is crepuscular. If we redefine our Activity index (described in Materials and Methods) as:

Activity index=Proportion of detections in daylight,dusk,night,or dawn period/Proportion of time in daylight,dusk,night,or dawn period

we find Activity Indices (mean ± *SD*) over the whole year of 0.55 ± 0.38 for daylight, 2.35 ± 0.53 for dusk, 0.99 ± 0.29 for night, and 1.60 ± 0.42 for dawn. Activity index differs between 24‐hr divisions (ANOVA *F*_3,44_ = 42.56, *p* < .001) with significant differences between all divisions (*p* < .004) except for the daylight/night comparison (*p* = .06). A figure above 1 indicates a high activity in relation to the time available in that time period, again consistent with a crepuscular behavior.

## 4 DISCUSSION

We suggest that there is a need for a consensus to be reached in the animal ecology community concerning the definitions of twilight, dusk, and dawn. Everyday usage defines twilight as the half‐light that follows sunset or precedes sunrise and ‘crepuscular’ as behavior that inhabits these time periods. However, the scientific literature is inconsistent on the definition of these terms in the context of diel activity. Many authors use a 2‐hr span that straddles sunset or sunrise (Caravaggi et al., 2018; Ikeda et al., 2016; Ogurtsov et al., 2018). Other authors (Campera et al., 2019; Narendra et al., 2010) define dusk and dawn in terms of astronomical twilight which ends or begins when the sun is 18° below the horizon (Thorsen, 2020). Still other authors (such as Bennie et al., 2014) use nautical twilight that ends or begins when the sun is 12° below the horizon (Thorsen, 2020). These variations in definition across studies means that it is not always possible to compare like with like.

We propose that the triggers of activity in species should be considered when defining twilight for biological studies. During postsunset and presunrise periods, the fall or rise in light intensity is exponential (Kishida, 1989; Thorsen, 2020). A wide range of animal species have been shown to alter behavior in response to these steep changes in light intensity (Leopold & Enyon, 1961; Narendra et al., 2010; Pepin & Cargnelutti, 1994). On that basis, we find no justification for defining twilight as the 2‐hr period straddling sunrise and sunset. Using that definition can affect interpretation of activity profiles. In the area of mountain hare research, Ikeda et al. (2016) and Ogurtsov et al. (2018) show postsunset and presunrise activity peaks that are offset because of their use of this definition of twilight.

In our analysis of activity patterns, we define the twilight periods as 2 hr after sunset and 2 hr before sunrise. Our preference would have been to use astronomical twilight which, at the latitude of the present study, is close to 2 hr for much of the year. However, in mid‐summer, the sun never reaches 18° below the horizon and the limit of astronomical twilight does not exist. Our 2 hr twilight periods are therefore a pragmatic solution and the 2 hr spans do match the width of the postsunset and presunrise activity peaks that we see (e.g., in Figure 4a).

There are two published studies on mountain hare activity using camera traps. They were conducted in forested areas in Hokkaido, Japan (Ikeda et al., 2016), and in the Valdai upland of Russia (Ogurtsov et al., 2018). Both use a definition of twilight as straddling sunset and sunrise but, taking that into account, their seasonal data show a bimodal crepuscular pattern with increased daylight activity in summer, a pattern that resembles our own results. However, the Ogurtsov et al. (2018) activity profiles for winter and spring followed a nocturnal activity pattern with a single peak in the middle of the night. This pattern differs from our own results and may be a consequence of the different habitats under study.

We have argued above that the triggers of activity in species should be considered when defining twilight for biological studies. The low light intensity of twilight stimulates secretion of the hormone melatonin from the pineal gland (Goldman, 2001). Melatonin determines activity levels and timings in crepuscular and nocturnal animals, and laboratory studies have demonstrated that this basic mechanism is subject to seasonal variations coupled to the reproductive cycle (Bartness & Goldman, 1989; Goldman, 2001). The Syrian hamster *(Mesocricetus auratus)* is a ‘long‐day breeder’ and Goldman proposed that, in January and February, the gonads of the Syrian hamster become fully active. Under the influence of the reproductive hormones, the animal enters a photorefractory phase. A similar timing of scrotal development in the male occurs in the mountain hare (also a ‘long‐day breeder’) and all female adults are pregnant between March and June (Angerbjörn & Flux, 1995). Thus, in mountain hare, we propose that the onset of daylight activity that we see in Spring may be associated with a partial suppression of the melatonin‐mediated circadian rhythm by the reproductive hormones. In the Syrian hamster (and we propose with mountain hare), this photorefractory phase ends with the shorter days as autumn approaches and that leads to regression of the reproductive organs, the restoration of full photosensitivity and the return to the autumn–winter pattern of crepuscular/nocturnal activity. This simple model based on the interaction of the reproductive hormones with the underlying circadian rhythm is likely to be subject to influence by other environmental factors, such as temperature, and physiological factors, such as nutrition.

We have argued above that our results can be interpreted as showing a basic crepuscular pattern that is modified during the breeding season leading to increased daylight activity. Although such activity represents a risk in terms of predation, it may be essential for efficient reproduction. In Scotland, the only raptors capable of taking an adult mountain hare are golden eagle (*Aquila chrysaetos*) or white‐tailed eagle (*Haliaeetus albicilla*) but neither are resident in the study area (this may change in the future with the recent reintroduction of the golden eagle to southern Scotland (https://www.goldeneaglessouthofscotland.co.uk/). Therefore, the risks to a prey animal of daylight activity in this specific area may be low. Would the diel activity pattern be different for animals living in unmanaged areas with greater predation pressure? It would be informative to carry out a diel behavioral study of mountain hares in the alpine areas of the Cairngorm Mountains of Scotland that are not managed for grouse and where there are aerial predators.

## 5 CONCLUSION

The data presented are the first of their type on the Lammermuir mountain hare population, indeed the first for southern Scotland. Despite the limited size of the camera trap study area (0.25 km^2^) and small number of camera trap locations (*n* = 5), the density and activity of mountain hares at the camera trap locations provided a large number of detections. This allows us to perform an informative analysis of patterns of circadian and seasonal activity. We find a greatly increased activity in daylight in spring and summer that is superimposed on the underlying crepuscular pattern present through the year. We suggest that the driver for seasonal change in activity is the reproductive cycle and the mechanism is hormonal change.

## CONFLICT OF INTEREST

None.

## AUTHOR CONTRIBUTIONS

**Graham W. Pettigrew:** Conceptualization (lead); data curation (lead); formal analysis (lead); funding acquisition (lead); investigation (lead); methodology (lead); project administration (lead); writing‐original draft (lead); writing‐review & editing (lead). **Valentina Di Vita:** Formal analysis (supporting); methodology (supporting); writing‐review & editing (supporting). **Maxine Pettigrew:** Formal analysis (supporting); writing‐review & editing (supporting). **Jason S. Gilchrist:** Formal analysis (supporting); methodology (supporting); writing‐original draft (supporting); writing‐review & editing (supporting).

## ETHICAL APPROVAL

The study has no ethical implications.

## Data Availability

Camera trap photographs of mountain hare Lepus timidus per month Scotland (S1a), https://doi.org/10.5061/dryad.m0cfxpp3p. Calculation of times relative to sunset and sunrise for mountain hare Lepus timidus camera trap records Scotland (S1b), https://doi.org/10.5061/dryad.2bvq83bpx. Calculation of detection rate for camera trap records of mountain hare Lepus timidus Scotland (S2), https://doi.org/10.5061/dryad.4b8gthtc4
